# Sinus Bradycardia after Extensive Neck Dissection and Total Thyroidectomy in a Pediatric Patient: A Case Report

**DOI:** 10.1055/s-0040-1721429

**Published:** 2020-12-14

**Authors:** Antonia Malli, Ioannis Skondras, Maria Vakaki, Alexander Passalides

**Affiliations:** 1 Department of B' Pediatric Surgery, General Children's Hospital of Athens “Panagiotis and Aglaia Kyriakou,” Attikis, Greece; 2 Department of Radiology, General Children's Hospital of Athens “Panagiotis and Aglaia Kyriakou,” Attikis, Greece

**Keywords:** papillary thyroid carcinoma, neck dissection, sinus bradycardia, postoperative

## Abstract

Postoperative complications after total thyroidectomy with extensive neck dissection in thyroid malignancies are well documented in the current literature. However, sinus bradycardia as a postthyroidectomy complication is a rare phenomenon and, to the best of our knowledge, few studies have identified it as a perioperative condition. In our study, we report a case of 9-year-old boy with papillary thyroid carcinoma, who underwent total thyroidectomy and bilateral neck dissection. Postoperatively, the surgery was complicated by initial vocal cord paresis and chyle leak. The patient also suffered from asymptomatic sinus bradycardia which self-resolved. Although causative factors cannot be determined by a single case, hypothyroidism, carotid sinus hypersensitivity, and bilateral damage to the middle cervical sympathetic ganglion could play a significant role in this uncommon pathophysiological condition.

Thyroid cancer in the pediatric population is a rare entity, representing approximately 5% of all head and neck malignancies, while the papillary type remains the most frequent neoplasm of the thyroid. 1 It is also well known that bilateral neck dissection and total thyroidectomy are considered challenging procedures with various complications such as recurrent laryngeal nerve injury, hypoparathyroidism, and chyle leak, among others, being commonly reported in the current literature. 1

Despite these well-described complications, sinus bradycardia has only been previously described as a rare perioperative phenomenon, to our knowledge. 2 The main aim of our study is to report such cases of persistent postoperative sinus bradycardia, which lasted several days. We present the case of a 9-year-old boy who suffered from sinus bradycardia after a total thyroidectomy combined with bilateral cervical lymphadenectomy.

## Case Presentation

**Fig. 1:**
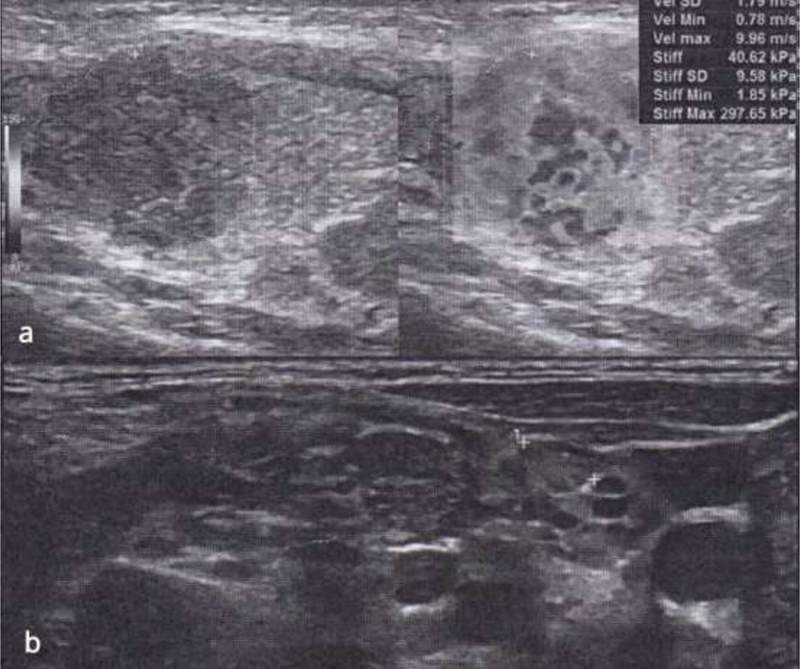
US of the neck and thyroid revealed ( **a** ) a mass in the right lobe measuring 2.57 × 1.61 × 2 cm with ill-defined margins, along with multiple calcifications and increased vascularity. The mass was located directly beneath the thyroid capsule, but whether or not it extended beyond the capsule was unclear. ( **b** ) Multiple nodules in the neck area, the largest being 4 × 1.5 cm in size. A nodule of 0.74 × 0.48 cm in size is shown in the right supraclavicular area (between *white crosses* ). All the above-mentioned nodules had heterogeneous characteristics, calcifications, increased vascularity, and poorly defined vascular hilum. US, ultrasound.

**Fig. 2:**
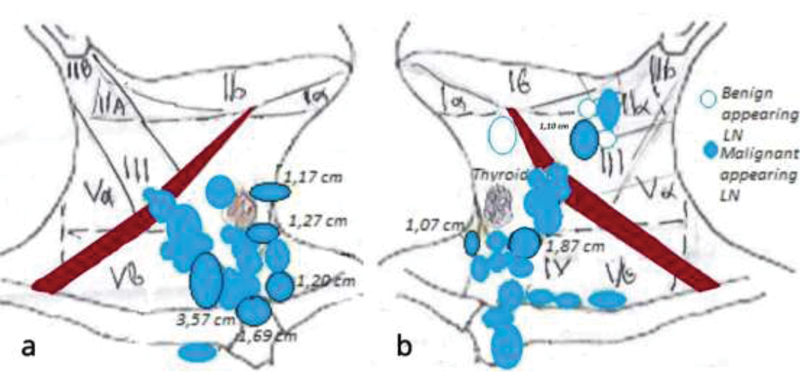
Mapping of lymph nodes. ( **a** ) Right lateral neck mapping shows multiple lymph nodes in different neck compartments with malignant characteristics with the largest in size ∼3.57 cm. ( **b** ) Left lateral neck reveals also multiple lymph nodes with both malignant and benign characteristics, with extension to the mediastinum.

A 9-year-old boy was admitted to our hospital after a routine physical examination that revealed a palpable thyroid and cervical mass. An ultrasound of the neck showed a mass in the right thyroid lobe measuring 2.57 × 1.61 × 2 cm with ill-defined margins directly beneath the thyroid capsule, along with multiple calcifications and increased vascularity. Multiple nodules were observed in the neck area, the largest being 4 × 1.5 cm in size ( Figs. 1 and 2 ). Fine needle aspiration of the mass and the cervical nodules revealed papillary cancer of the thyroid (Bethesda VI) that was also present in the nodules. Preoperative laboratory studies showed a normal value of thyroid-stimulating hormone and free T4, whereas thyroglobulin had risen to 913.5 ng/mL (normal range: 5.80–37.40 ng/mL).

Based on the above findings, a total thyroidectomy combined with bilateral modified neck dissection with removal of the lymph nodes at levels II, III, IV, V, VI, and VII was performed. Three of the parathyroid glands were also removed after being accidentally devascularized. Intraoperative nerve monitoring did not reveal any nerve injury. Finally, two paratracheal redopack drains were used.

A few hours after surgery, the patient displayed respiratory distress lasting a few minutes and emergent laryngoscopy also revealed unilateral vocal cord paresis and edema with subglottic extension. For this reason, the patient was then intubated and remained in the intensive care unit (ICU) for 5 days. While in the ICU, sinus bradycardia of a heart rate (HR) 55 to 60 bpm (normal HR: 80–120 bpm) and premature ventricular beats were newly noted on the electrocardiogram.

Subsequently, after his successful extubation and an immediate postextubation laryngoscopy which revealed normal vocal cord mobility, the patient was transferred to the pediatric surgery department where he was on continuous cardiac monitoring. Asymptomatic sinus bradycardia persisted along with premature ventricular beats. Cardiac ultrasound revealed no heart abnormalities with normal ejection fraction. At postoperative day 2, chylous fluid started draining at a rate of 100 mL per day from both drains and thus, thoracic duct injury was suspected. This complication was managed conservatively with total parenteral nutrition and administration of somatostatin for 17 days, with a slow but significant decrease in the output of the drains. The patient started fat-free diet per mouth at postoperative day 20 and by postoperative day 27, the lymphatic leak and sinus bradycardia had also resolved. Overall, chyle leak lasted 25 days and the drains were removed at postoperative day 27. No electrolyte disturbances were noticed and the calcium levels (ionized and total) were ranging between 8.5 and 9.2 mg/dL (normal values: 8.5–10.2 mg/dL) throughout his hospital stay. Almost all of the lymph nodes (>10) excised tested positive for tumor infiltration in the histopathological study, except for three lymph nodes in the left III level of neck dissection. The patient was finally discharged from our hospital for further management and oncologic assessment.

## Discussion

A search in PubMed, EMBASE, and Google Scholar revealed no similar phenomenon of sinus bradycardia, neither in pediatric nor in adult cases of postthyroidectomy complications.

Several potential mechanisms could be hypothesized to explain this rare case of arrhythmia. First, the hypothyroidism observed in the immediate postoperative period due to total thyroidectomy and the gradual replacement with thyroid hormones led to some cardiovascular alterations. Sinus bradycardia is a common cardiovascular event seen in hypothyroidism and is usually caused by sinus node dysfunction. 3 In general, several studies have shown that triiodothyronine (T3) plays the primary role in heart function by regulating the speed of systolic contraction and diastolic relaxation, 3 mediated by the action of a nuclear thyroid receptor (TR) with two isoforms, TRα and TRβ. T3 has inotropic and chronotropic effects by binding to the predominant nuclear receptor of the heart, TRα. 3 Consecutively, a short period of hypothyroidism following the abrupt loss of thyroid hormones and function due to total thyroidectomy could be a possible cause of the sinus bradycardia in the short-term postoperative period. The slow replenishment of these hormones and the normalization of their effect on peripheral tissues could explain the fact that the sinus bradycardia resolved without any medication.

Another possible pathophysiological mechanism in this case could be the carotid sinus hypersensitivity. It is well documented that head and neck malignancies can produce mainly perioperatively negative chronotropic and inotropic cardiac events. 4 Carotid sinus hypersensitivity has been proposed as the main cause for this particular phenomenon, as injury to the sinus either by compression and/or invasion by the tumor and lymph nodes or mechanical injury during surgery could lead to exaggeration of the effect of carotid sinus. 4 There are three types of carotid type hypersensitivity that are reported in the current literature: the first type or the cardioinhibitory type is being predominantly expressed with negative chronotropic effect, resulting in sinus bradycardia. The second type, the vasodepressor, which is less common than the first one, is expressed with hypotension. The third type is the mixed type, which can be manifested with both hypotension and bradycardia, but remains very rare. 4 Our patient's sinus bradycardia could be explained by the cardioinhibitory effect of the carotid sinus exaggeration due to potential transient mechanical injury during the extensive neck dissection and/or by thermal injury during surgery.

Last but not least, this decrease in HR could also be attributed to the damage of the middle cervical sympathetic ganglion (MCG) intraoperatively. Several studies have shown that MCG plays a significant role in the thoracic sympathetic innervation in normal hearts, especially in the regulation of HR 5 . It is usually located at the level of C5–C7, typically anterior to the longus colli muscle, but exhibits many anatomical variations that must be taken into consideration. 5 In some cases, the cervical sympathetic chain can pass within the posterior wall of the carotid sheath. Its anatomic variability, lymph node-like small size, and oval shape render it susceptible to injury during neck surgery, often caused by thermal injury, aggressive dissection, or even lateral retraction of the carotid artery. 5 Bilateral MCG damage during extensive neck dissection could subsequently lead to bradycardia.

Hypothyroidism, carotid sinus stimulation, and bilateral damage to the middle cervical ganglion could contribute to possible causes of sinus bradycardia. Thus, more studies need to be conducted in an experimental and clinical setting to assess the validity of our hypotheses.

## Conclusion

While no causative factors can be derived from a single case report, further studies should assess the necessity of cardiac monitoring in the immediate postoperative period in cases of extended neck dissection and thyroidectomy, so that such cardiovascular complications can be properly assessed and managed.
